# Prospective Pharmacological Potential of Resveratrol in Delaying Kidney Aging

**DOI:** 10.3390/ijms22158258

**Published:** 2021-07-31

**Authors:** Md Jamal Uddin, Mithila Farjana, Akhi Moni, Khandkar Shaharina Hossain, Md. Abdul Hannan, Hunjoo Ha

**Affiliations:** 1 ABEx Bio-Research Center, East Azampur, Dhaka 1230, Bangladesh; mithilafarjanabge@gmail.com (M.F.); akhimoni840818@gmail.com (A.M.); kshrumky2010@gmail.com (K.S.H.); hannanbmb@bau.edu.bd (M.A.H.); 2 Graduate School of Pharmaceutical Sciences, College of Pharmacy, Ewha Womans University, Seoul 120-750, Korea; 3 Department of Biochemistry and Molecular Biology, Bangladesh Agricultural University, Mymensingh 2202, Bangladesh

**Keywords:** antioxidant, anti-aging, AKI, CKD, kidney aging, resveratrol

## Abstract

Aging is an unavoidable part of life. The more aged we become, the more susceptible we become to various complications and damages to the vital organs, including the kidneys. The existing drugs for kidney diseases are mostly of synthetic origins; thus, natural compounds with minimal side-effects have attracted growing interest from the scientific community and pharmaceutical companies. A literature search was carried out to collect published research information on the effects of resveratrol on kidney aging. Recently, resveratrol has emerged as a potential anti-aging agent. This versatile polyphenol exerts its anti-aging effects by intervening in various pathologies and multi-signaling systems, including sirtuin type 1, AMP-activated protein kinase, and nuclear factor-κB. Researchers are trying to figure out the detailed mechanisms and possible resveratrol-mediated interventions in divergent pathways at the molecular level. This review highlights (i) the causative factors implicated in kidney aging and the therapeutic aspects of resveratrol, and (ii) the effectiveness of resveratrol in delaying the aging process of the kidney while minimizing all possible side effects.

## 1. Introduction

The aged population is expanding exponentially worldwide [1]. Recently, healthy aging has been regarded as a critical issue due to the increase of the elderly population. The risk of kidney disease among the elderly has been increasing. Kidney aging is associated with age-related comorbidities. While the detailed molecular mechanism underlying kidney aging is not yet known, acute kidney injury (AKI) and chronic kidney disease (CKD) share many phenotypic similarities with aging, including cellular senescence, inflammation, fibrosis, vascular rarefaction, loss of glomeruli, and tubular dysfunction [2,3]. Interruption of the cell cycle leads to the accumulation of senescent tissues in multiple organs, including the kidney, with advancing age [4]. The stable transformation of the kidney structure and its functions in the elderly exhibits a significant reduction in the number of glomeruli (responsible for nephrosclerosis), cortical volume, glomerular filtration rate (GFR), and manifold kidney cysts [3,5,6]. Premature aging and a high mortality rate are exacerbated by inflammation leading to multiorgan dysfunction or diseases [7]. Thus, the mechanisms associated with kidney diseases may help in understanding the molecular pathways involved in kidney aging. At the same time, a promising therapeutic regimen is urgently needed to normalize age-associated issues, including kidney aging [3].

Resveratrol (3,4′,5-trihydroxy-trans-stilbene), a versatile phenolic compound found in various fruits, especially red grapes and vegetables, possesses anti-aging effects and thereby can promote life span through modulation of the important hallmarks of aging, for example, inflammation, oxidative stress, and fibrosis, angiotensin II (Ang II), cell senescence, telomere attrition, mitochondrial dysfunction, angiogenesis, and platelet aggregation [8,9,10,11]. Resveratrol tends to restrain cells from aging and inhibits senescence-associated secretory phenotype development [12]. Calorie restriction (CR) and resveratrol possess similar anti-aging properties by extending the lifespan [13]. Additionally, in a cellular senescence model of IMR-90 cells, 10 μM of resveratrol more effectively decreased cellular senescence and apoptosis than CR [13]. These findings indicate that resveratrol could be a potential alternate anti-aging therapy.

Furthermore, more than 244 clinical trials, along with 27 ongoing experiments, have investigated the safety and efficacy of resveratrol along with its pleiotropic functions. It is reported as safe at up to 5 g/day doses and shows therapeutic potential against various cancers, diabetes, obesity, hypertension, cardiovascular diseases, kidney diseases, inflammatory diseases, Alzheimer’s disease, and so on [14,15,16]. Nihei and colleagues reported that oral resveratrol treatment improves clinical parameters of nephrotic syndrome, including proteinuria and hypoalbuminemia, and normalized dyslipidemia in rats [17]. Resveratrol is shown to attenuate AKI by regulating antioxidant and anti-inflammatory mechanisms in rats [18,19]. Recently, health benefits of resveratrol for treating various kidney diseases have been reviewed [20,21,22]. Considering its pharmacological potential across the body systems and diverse conditions, here we mainly discuss the prospects of resveratrol in the therapeutic management of kidney aging and its associated abnormalities.

## 2. Methods

A search of the literature was carried out to collect published research information on the effects of resveratrol on kidney aging from available online databases, such as PubMed, Google Scholar, and Scopus, using the keywords ‘resveratrol on kidney diseases’ and ‘resveratrol on the aging process in the kidney such as telomere shortening, DNA damage, cellular senescence mitochondrial damage, endoplasmic reticulum stress (ER stress), autophagy dysfunction oxidative stress, inflammation, fibrosis, lifespan extension, calorie restriction, and epigenetic modulation’. All figures were generated using BioRender.com (accessed on 31 July 2021). 

## 3. Pharmacological Effects of Resveratrol on Kidney Diseases

Patients who have a history of AKI may develop progressive CKD [23]. CKD is characterized by mitochondrial damage, ER stress, autophagy dysfunction, oxidative stress, inflammation, and fibrosis. The protective effects of resveratrol on various kidney diseases, such as AKI and CKD, in vitro and in vivo have been reported [20] and now are summarized below.

### 3.1. Acute Kidney Injury

AKI is a common kidney disease generally associated with an increased fatality rate [24,25]. Reduced glomerular function and urine output are the primary signs of AKI [26]. The severity of this disease can be estimated through a study demonstrating that an alarmingly high percentage of people (16.9% to 31.0% in Western countries) are suffering from this condition [27]. 

Many studies have shown that compounds, such as resveratrol, can provide us with a wide range of therapeutic options to act against various factors that are linked to different diseases [28,29]. In humans, resveratrol has proven its potency against AKI by inhibiting the formation of reactive oxygen species (ROS) [30]. Resveratrol-loaded nanoparticles could prevent ischemia/reperfusion (I/R)-induced kidney injury in a rat model [31]. Another study on rabbits showed that resveratrol reduced kidney hypoxia, mitochondrial dysfunction, and kidney tubular cell apoptosis [32]. Resveratrol has also been found to be an effective agent to downregulate tumor necrosis factor-α (TNF-α), interleukin (IL)-1β, and kidney injury molecule (KIM)-1 expression in AKI [33]. Resveratrol could decrease the mortality rate of septic rats and alleviates AKI by relieving ER stress, inhibiting NF-κB pathway activation, and mitigating the inflammatory response [34]. All of these studies indicate that resveratrol can be a potential agent to fight against AKI. An in vitro study showed that resveratrol was shown to increase cell viability while reducing phosphorylation of nuclear factor-κB (NF-κB) and the production of inflammatory factors in response to lipopolysaccharide (LPS), and reduced damage to tunicamycin-induced human kidney 2 (HK-2) cells through inhibiting inositol-requiring enzyme 1 (IRE1) activation [35]. While resveratrol reduced cadmium-induced mitochondrial ROS and apoptosis, it increased mitochondrial biogenesis as well as cell viability in TCMK-1 renal epithelial cells [36]. In addition, resveratrol decreased ochratoxin A (a nephrotoxin)-induced intracellular ROS production and cellular damage in HEK293 cells [37]. However, a detailed investigation is required to elucidate a precise mechanism of resveratrol action in AKI.

### 3.2. Chronic Kidney Disease

CKD is known to alter the regular function and structure of the kidney, usually irreversibly [38]. Several conditions, such as diabetes, hypertension, proteinuria, reduced cytokine clearance, and chronic infections, are the most common risk factors for CKD [39,40,41]. Resveratrol has been shown to increase the expression of muscle ring-finger 1 (MuRF1) and inhibit the phosphorylation of NF-κB in an in vivo model of CKD [42]. There is a link between mitochondrial dysfunction and CKD pathogenesis. Resveratrol was shown to play a significant role in the recovery of CKD by improving mitochondrial function through a mechanism that involved the preservation of mitochondrial membrane potential loss, enhancing the ATP level, reducing ROS generation, and facilitating oxidative phosphorylation in nephrectomized rats [43]. Resveratrol nanoparticles could be a better candidate for preventing CKD through attenuation of the NLR family pyrin domain containing 3 (NLRP3) inflammasome [44]. 

Individuals suffering from CKD may experience a reduced function of antioxidant defense due to reduced consumption of antioxidant vitamins and minerals, such as vitamin C and selenium. Due to its antioxidant capacity, resveratrol has the ability to fight CKD [45,46]. Consumption of white wine and olive oil together reduced CKD plasma biomarkers, suggesting a possible anti-inflammatory effect on CKD patients [47]. One study reported that resveratrol effectively reduced the degree of kidney tubular damage in unilateral ureteral obstruction-induced fibrotic rats [48]. Pterostilbene, an analog of resveratrol, prevents renal fibrosis and epithelial to mesenchymal transition (EMT) in mice [49]. All of this evidence suggests that resveratrol can act as a potential agent against CKD.

In HK-2 cells, resveratrol reduced high glucose-induced oxidative stress (such as MDA and ROS levels) by increasing superoxide dismutase (SOD) and catalase [50]. Additionally, resveratrol reduced the transforming growth factor-β (TGF-β)-induced EMT in HK-2 cells in a dose-dependent manner [51]. Resveratrol decreased the cisplatin-induced cellular injury and apoptosis in mouse proximal tubular cells [52]. Resveratrol inhibited oxalate-induced inflammatory cytokines, colonization, and hyaluronan protein level while it increased several antioxidant enzyme activities in human renal epithelial cells [53]. 

Platelet-derived growth factor (PDGF) is a potent stimulus for mesangial cell proliferation and involved in the pathogenesis of glomerulonephritis [54]. Treatment of mesangial cells with resveratrol inhibited PDGF-induced cell proliferation by regulating PI3K, Akt, ERK1/2, and c-Src [54]. In podocytes, resveratrol reduced high glucose-induced mitochondrial stress, mitochondrial ROS production, mitochondrial dysfunctions, and apoptosis [55]. In HEK293 cells, resveratrol reduced high glucose-induced aging markers, such as β-galactosidase, while it increased SIRT1 and thioredoxin [56]. 

However, long-term (72 h) exposure to high doses (40–80 μM) of resveratrol increased mitochondrial ROS, fibrotic, and apoptotic protein levels, while it reduced anti-apoptotic proteins and mitochondrial function in HK-2 cells [51]. Since higher ROS levels cause a ROS burst leading to mitochondrial damage [57], high-dose resveratrol-induced higher ROS levels in mitochondria may increase mitochondrial damage in the cells. Additionally, resveratrol at high doses (50–75 µM) increased NF-κB-mediated inflammatory effects in IL-1β or TNF-α-treated mesangial cells and kidney proximal tubular LLCPK1 cells [58]. However, considering the critical doses of resveratrol to induce either protective or cytotoxic effects, substantial research is needed to rule out the controversial effects caused by resveratrol treatment and to recommend for clinical use.

## 4. Pharmacological Effects of Resveratrol on Aging Biomarkers in the Kidney

**Figure 1:**
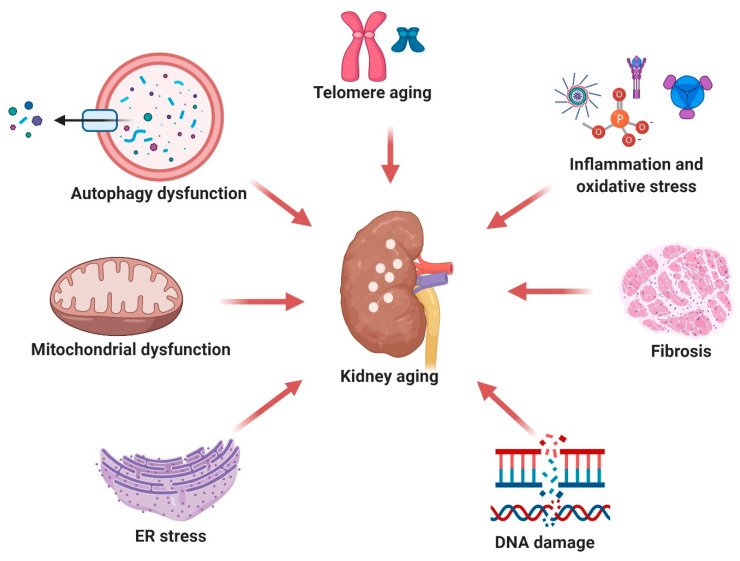
Pathological features of the kidney aging process. Kidney aging mainly occurs through several events in a sequential manner. These include (i) kidney fibrosis along with a reduction in cortical mass, increased glomerulosclerosis, and promptness of RAS; (ii) cellular senescence occurs through a persisting DNA damage response and an increase in interferon-gamma (IFN-γ); (iii) mitochondrial damage induces an increased level of ROS through, which causes dysfunction with mitochondria; (iv) inflammation and oxidative stress are mediated through an increase in lipid peroxidation, NF-κB activation, and glutathione depletion; (v) ER stress; (vi) autophagy dysfunction; and (vii) telomere shortening occurs by limiting transcription by DNA polymerase, leading to GFR diminution, decreased urinary concentration, reduced urinary acidification, impaired potassium clearance, increased vascular resistance, a contracted kidney mass, and blood flow.

Resveratrol possesses a vast range of benefits in various organs, including the brain, liver, heart, lung, and pancreas, conferring anti-aging landmarks [14,15,16] (shown in Table 1). It also possesses various anti-aging landmarks in the kidney [20,21,22] (shown in Table 2). The anti-aging properties of resveratrol have been reviewed [59]. A growing body of evidence suggests that resveratrol exerts its protective effects against kidney aging by regulating the renin-angiotensin system (RAS) and alleviating inflammation, oxidative stress, and cellular senescence [10,11]. Health benefits of resveratrol on kidney epithelial cells, kidney corpuscle, kidney fibroblasts, and kidney cancer cells, even at the molecular level, both in vivo and in vitro, have been reported [20]. The pharmacological potential of resveratrol against some plausible factors (such as telomere shortening, cellular senescence through DNA damage, mitochondrial dysfunction, ER stress, and autophagy dysfunction oxidative stress, inflammation, and fibrosis, as shown in Figure 1) responsible for kidney aging are delineated in this section. 

### 4.1. Telomere Shortening 

The 6-bp recurred sequence, TTAGGG, which is known as the telomere, constructs the end of each mammal’s chromosome. Mitotic cell division is a fundamental process, and during each cycle of division, almost 50–200 bp telomeric sequences tend to be eroded due to the ″end replication problem″, causing telomere shortening [89]. The mechanism of limiting transcription by DNA polymerase is associated with replicative senescence, apoptosis, cancer, and CKD [90,91]. Thus, the telomeric length is considered to be a probable biomarker of kidney aging [92]. The most consolidated telomerase function having unlimited replicative dynamics is demonstrated in human cancers [91,93]. Telomeric shortening is ultimately responsible for significant changes in the kidney, such as decreases in GFR, urinary concentration, urinary acidification, kidney mass, and blood flow [94,95]. A critically short telomere evokes cell-cycle inhibitor p21 and CDKIs, which play a crucial role in arresting the cell cycle progression, resulting in apoptosis of kidney cells [96,97]. An inhibitory protein named p16INK4a is responsible for inhibiting the activity of CDK4 and CDK6 [96]. The manifestations of p21 and p16INK4a become exacerbated by activation of p53, with serious impacts on vital organs, such as the kidneys, in the elderly population [98]. The propensity to shortening of the telomeres is commensurate to kidney aging, along with aging in most other organs, tissues, and cells, including the lung, pancreas, liver, muscle, hepatocytes, intestinal epithelial cells, peripheral blood cells, lymphocytes, and vascular endothelial cells [99,100].

Telomerase is a reverse transcriptase enzyme (TERT) that adds repetitive sequences to telomeres in dividing cells to prevent the telomeres from shortening [101]. In CKD patients, the lowest levels of telomerase activity (TLMA) and TERT expression were detected, while they had the highest IL-6 and C-reactive protein (CRP) levels [102]. Resveratrol activates telomerase activity in epithelial [103] and endothelial progenitor cells [104]. Direct modulation of p53 by resveratrol has also been observed [52]. Resveratrol treatment promoted p53 deacetylation and thereby attenuated cisplatin-induced kidney apoptosis and improved the GFR [52]. 

However, in cancer, most cancer cells with limitless proliferative capacity protect their telomeres by expressing high TLMA. Resveratrol at higher concentrations (>2.5 µg/mL) induced a substantial and concentration-dependent downregulation of TLMA in carcinoma cell lines, with 100% inhibition at 40 µg/mL [105]. Besides, resveratrol was also shown to be effective in downregulating the expression of h-TERT protein in human A431 epidermoid carcinoma cells [106]. 

### 4.2. Cellular Senescence and DNA Damage 

Aberrant accumulation of chronic senescent cells in response to prolonged signaling promotes kidney disease is also linked to age-related declines in kidney function [107]. Cellular senescence is a state where cellular differentiation and proliferation are inhibited. DNA damage is a ubiquitous mediator for both replicative superannuation that is brought about by premature cellular senescence and telomere shortening induced by various pathogenic factors, such as oxidative stress, mutations, and the failure of DNA repair mechanisms [108]. In the kidney, both G1- and G2-arrested senescent cells accumulate with advancing age and kidney disease [4]. A persistent DNA damage response (DDR) signal is provided by senescent cells to trigger the targeted arrest. The activation of DDR affects the chromatin of damaged DNA and the whole genome as well [109]. Moreover, the pathogenesis of atherosclerosis becomes exacerbated by interferon-gamma (IFN-γ), which entails the promotion of rapid cellular senescence following triggering of a p53-dominated DNA damage pathway [110]. Oxidative stress acts as a catalyst in the cellular senescence process, and this situation leads to kidney damage, which eventually turns into kidney aging. 

Sepsis is a state of disrupted inflammatory homeostasis causing multiorgan failure, where resveratrol treatment is intended to alleviate oxidative DNA damage [78,111]. Resveratrol treatment attenuates the dysregulation of regulators involved in the cell cycle and senescence pathways [112]. Additionally, resveratrol treatment impedes high-glucose-induced cell senescence (β-galactosidase) in the kidneys [113]. Resveratrol administration resulted in dysregulation of regulators involved in cell cycle and senescence pathways, such as cyclin-dependent kinase (CDK4 and 6), cyclin D1, p21, and p16, leading to senescence instead of apoptosis in gastric cancer in vivo and in vitro [112]. 

### 4.3. Mitochondrial Damage 

Mitochondria are considered highly dynamic organelles because they have numerous functions within a cell [114]. Mitochondrial damage has a significant impact on kidney function. Although mitochondrial ROS plays a protective role up to a certain level, it can cause mitochondrial dysfunction and cellular damage after exceeding the limit [115]. The mutation rate of mitochondrial DNA (mtDNA) is 10 to 1000 times higher than that of chromosomal DNA. Both exogenous (UV, base analog, ROS, and others) and endogenous stimuli (single or double strand breakdown, mismatch in base pair) may cause mtDNA damage [116]. Thus, a potential therapeutic option that can improve mitochondrial fitness is necessary to treat kidney diseases [117]. 

Resveratrol preserves mitochondrial integrity and enhances autophagy through inhibition of mitochondria damage signals like NLRP3 inflammasome-derived IL-1β production and pyroptosis in macrophages [84]. Resveratrol supports the escape of mtDNA from ROS by reducing cellular H_2_O_2_ and NO levels [118,119]. Resveratrol increases the expression of proteins involved in the electron transport chain, the mitochondrial content, and mitochondrial biogenesis markers [118]. In addition, resveratrol was shown to activate autophagy to attenuate mitochondrial dysfunction and apoptosis [73]. Administration of resveratrol improves mitochondrial function in kidneys through upregulation of sirtuin type 1 (SIRT1) and PGC-1α deacetylation, which constitute one of the most significant kidney protective mechanisms of resveratrol [43].

### 4.4. ER Stress 

A role of ER stress is common to several kidney disease conditions, including kidney fibrosis, glomerulopathies, primary glomerulonephritis, diabetic nephropathy, and so on. ER stress induces apoptosis of kidney cells, leading to kidney damage [120,121,122]. Glycation stress is a contributing factor that involves an imbalance of protein homeostasis and post-translational modification of proteins in the kidney that leads to ER stress [123]. Even slightly diminished autophagy or micro-autophagy accelerates inflammation and ER stress in adipose tissue with aging [124]. The mechanism involved in ER stress in the kidney is related to a complicated chain/pool of stress signaling networks, where several parameters, such as oxidative stress, the Akt pathway, and lipid and epigenetic alterations, make the kidney susceptible to hypoxia stress [125]. Moreover, redox signaling mechanisms involved in ROS cascades become exacerbated by ER stress, which not only causes kidney injury but also results in multiple disorders in humans [126].

Looking for a solution, due to limited mechanism-based therapies targeting kidney diseases/aging, modulation of ER stress using pharmacological regimes could be a promising option/approach [121,127]. Apart from this, knockdown of ER stress-expressing proteins, for instance, reticulon 1 (RTN1), could contribute to halting the stress along with minimizing kidney risks [128]. Moreover, resveratrol, as a natural treatment, demonstrated a beneficial effect in alleviating ER stress and thereby improving kidney function and tubular cell injury [35]. Resveratrol has been proven to reduce the levels of ER stress-related factors, which in turn attenuate urinary protein, blood glucose, kidney damage, and AKI along with the mortality rate [34]. Resveratrol even protects against cadmium (Cd)-induced ER stress and nephrotoxicity [129]. Furthermore, H_2_O_2_-induced ER stress is also alleviated by treatment with resveratrol by improving the redox balance in bovine mammary epithelial cells [130]. 

### 4.5. Autophagy Dysfunction 

Autophagy is crucial for protein homeostasis [121]. Though oxidative stress and autophagy dysfunction are intimately involved in kidney diseases, very little is known about the signaling processes that link them [131]. Autophagy is a degradation pathway of lysosomal proteins in a highly regulated manner, where ablation of protein aggregates and damaged organelles occurs to maintain intracellular homeostasis and cellular integrity, and therefore, an aberration of this pathway leads to the pathogenesis of a variety of kidney diseases and aging [132,133]. For instance, impairment/disturbances in autophagic flux may lead to pathogenesis of kidney lipotoxicity, kidney injury, lysosomal dysfunction, AKI, diabetic nephropathy, focal segmental glomerulosclerosis, polycystic kidney disease, and kidney aging [134,135,136]. However, the induction of autophagy during CKD in mice is responsible for the impairment of mitochondria and ATP production [137].

Activation of autophagy plays an important role against kidney diseases and the aging process, while sirtuins, mammalian target of rapamycin (mTOR), and AMP-activated protein kinase (AMPK) are the key regulators of autophagy [134,138]. Resveratrol inhibits the NLRP3 inflammasome pathway through induction of AMPK and SIRT1-mediated autophagy [139]. The effects of resveratrol on autophagy were shown against diabetic nephropathy, where this compound enhanced the LC3-II/LC3-I protein ratio and downregulated cleaved caspase-3 expression [81]. Long-term use of resveratrol is reported to reduce type 2 diabetic nephropathy since it promotes SIRT1-mediated autophagy induction [82]. Additionally, in vitro and in vivo studies showed that resveratrol reduced oxalate-induced kidney inflammation and oxidative stress through autophagy activation [140].

Pterostilbene, an analog of resveratrol, has several health benefits through activation of autophagy. Pterostilbene prevents kidney fibrosis via activation of autophagy and attenuation of the NLRP3 inflammasome and EMT [49]. Additionally, resveratrol nanoparticles induce autophagy and inhibit CKD through inhibition of the NLRP3 inflammasome and IL-1β production [44]. 

### 4.6. Oxidative Stress and Inflammation 

Oxidative stress and inflammation are two common pathogenic factors that account for functional changes in the kidney during senescence. Oxygen-free radicals and pro-oxidants serve as an exaggerating player in both CKD and AKI [141,142]. CKD/aging, exacerbated by both oxidative stress and inflammation, involves a significant decline in SOD and glutathione peroxidase (GSH-Px) and a sudden increment of several pro-inflammatory cytokines, for instance, IL-1, IL-6, and TNF-α, along with CRP and MDA [143,144]. Excessive production of NO is triggered by inflammation-incited NO synthase, which exaggerates highly reactive superoxide radical generation. Furthermore, the reaction between excess NO synthase and SOD results in peroxynitrite formation. Besides, increased ROS and RNS elicit more age-provoking factors, for example, angiotensin II, chemokines, and so on [145]. The adverse effects exerted by reactive oxygen and nitrogen species (RONS) are obstructed by antioxidant defenses, and any inconsistency between them results in oxidative stress, and eventually tissue damage with further age-related complicacy [146]. The premature kidney aging phenotype incorporates muscle wasting, vascular calcification, depression, osteoporosis, and frailty, which are accelerated by systemic inflammation. On the other hand, uremic inflammation is engaged to alter the functional mechanism of the mitochondria, telomeres, and nutrient sensing [7]. The effects of oxidative stress and inflammation in the kidney provoke higher lipid peroxidation, NF-κB activation, and glutathione depletion, leading to Nrf2 deactivation and impaired antioxidant defenses [147]. 

As a forthright antioxidant, resveratrol serves as a shield for cellular biomolecules against oxidative damage by scavenging diverse RONS and secondary radicals [148]. Resveratrol was found to lower oxidative stress by activating the Nrf2 pathway [83,149], alleviating kidney inflammation and injury, recuperating the antioxidant capacity by promoting glutathione-S-transferase (GST) activity, mitigating hypertension, reducing apoptosis, and suppressing the NF-κB pathway along with caspase cascades [70,83,150]. Resveratrol treatment also exhibited its kidney protective effects by inhibiting inflammatory cytokines [151]. Resveratrol was proven to impede the manifestation of TNF-α and IL-1β in the hippocampus [152]. SIRT1 mRNA expression flourished, and age-related pro-inflammatory and pro-oxidant status was reduced in response to resveratrol treatment [70]. Additionally, a straightforward suppression of pro-inflammatory cytokines and, at the same time, the promotion of anti-inflammatory cytokine (IL-10) release by resveratrol were conspicuously exhibited [153]. The simultaneous prevention of inflammation and the disruption of endothelial cell permeability of kidney tissues by resveratrol significantly improved kidney function [77]. The blockage of NF-κB reduces pro-inflammatory factors (MCP-1, TNF-α, and CFB), and resveratrol alleviated inflammation in polycystic kidney disease [85]. Resveratrol can exert amazing renoprotection effects by inhibiting inflammatory responses and lowering oxidative stress via the Nrf2/TLR4/NF-κB pathway [150]. Additionally, resveratrol stimulates the induction of SOD expression and increases the GSH level while downregulating MDA and TNF-α [78]. Resveratrol treatment impedes high glucose-induced kidney oxidative stress by activating SIRT1 [113].

### 4.7. Fibrosis 

Kidney aging is considerably associated with a reduced cortical mass and GFR, and with glomerulosclerosis, tubular atrophy, interstitial fibrosis, and arteriosclerosis [154,155]. EMT is the main mechanism of kidney fibrosis, while the TGFβ-1-SMAD pathway and hypoxia are known as the main modulator of EMT [156,157]. Kidney tubular EMT is a major contributing factor to age-related kidney fibrosis. Additionally, the activation of RAS causes kidney fibrosis. In particular, Ang II and its corresponding receptors are responsible for mediating kidney injury, while angiotensin 1-7 has a protective role by counteracting the effects of Ang II [10]. 

Resveratrol is an effective therapeutic agent against diabetic kidney fibrosis [79]. Additionally, resveratrol restrains Ang II expression and exaggerates Ang 1-7 with improved kidney histologic findings both in vivo and in vitro [10,158]. Anti-fibrotic or pro-fibrotic kidney effects with improved kidney function were demonstrated upon resveratrol administration in a dose-dependent manner [51]. As matrix metalloproteinase K (MMP) is a promotional player in aging, identifying an agent that can block its expression is worthwhile. Resveratrol may attenuate kidney injury and fibrosis through inhibition of EMT [51]. Additionally, pterostilbene prevents renal fibrosis and EMT in high adenine diet-induced CKD mice [49]. By targeting both EMT and fibroblast–myofibroblast differentiation (FMD), resveratrol may successfully impede fibrosis formation and the myofibroblastic phenotype by suppressing the activity of the proliferation-related signaling pathways, such as MAPK, PI3K/Akt, and SMAD2/3 [87]. Resveratrol also alleviates age-related EMT in aging kidneys [159]. 

## 5. Effect of Resveratrol on Age-Related Mechanisms

**Figure 2:**
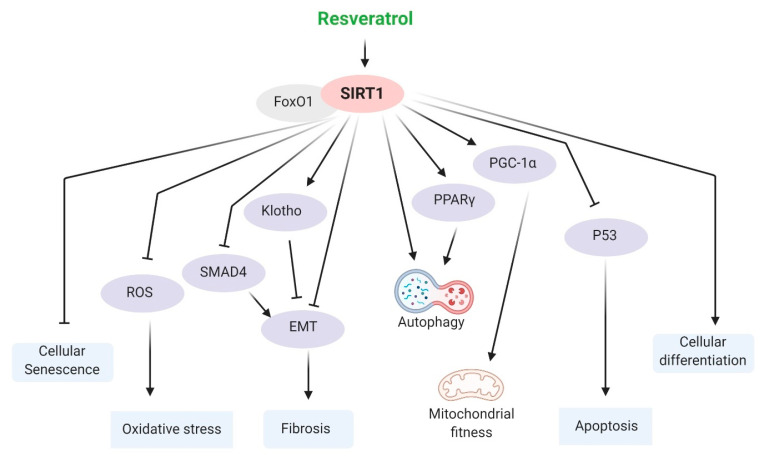
Resveratrol-induced SIRT1 is associated with a modulation of various mechanisms of age-related pathologies. Resveratrol stimulates binding between FoxO1 and SIRT1, and it may reduce kidney damage, myocyte hypertrophy, cellular senescence, oxidative stress, tubular apoptosis, and interstitial fibrosis. It also stimulates cellular differentiation and autophagy through SIRT1 activation. All of these pathways are involved in the higher potency of resveratrol against the aging process. Arrow means activation, and ‘T’ arrow means inhibition.

**Figure 3:**
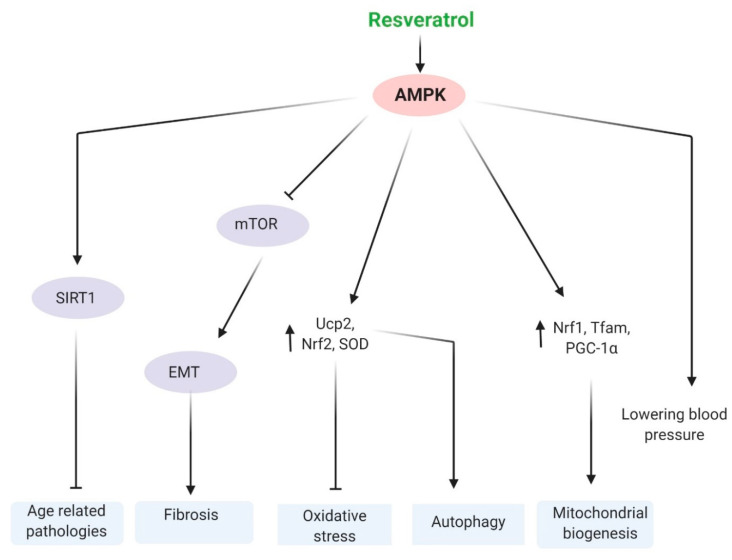
Resveratrol-induced AMPK is associated with the modulation of various mechanisms of age-related pathologies. Resveratrol-induced AMPK activates SIRT1, leading to the inhibition of many age-related pathologies. AMPK suppresses EMT and fibrosis through inhibition of mTOR. Nrf2, and SOD expression, which is associated with the activation of autophagy and the suppression of oxidative stress. Additionally, AMPK lowers blood pressure and increases mitochondrial biogenesis-related genes, such as Nrf1, Tfam, and PGC-1α. All of these pathways mediated by resveratrol-induced AMPK are associated with the anti-aging process. AMP-activated protein kinase, AMPK; epithelial–mesenchymal transition, EMT; sirtuin type 1, SIRT1; mammalian target of rapamycin, mTOR; mitochondrial uncoupling protein2 (UCP2); nuclear factor erythroid 2–related factor 2 (Nrf2); peroxisome proliferator-activated receptor-γ coactivator-1α, PGC-1α; and mitochondrial transcription factor A, Tfam. Arrow means activation, and ‘T’ arrow means inhibition.

**Figure 4:**
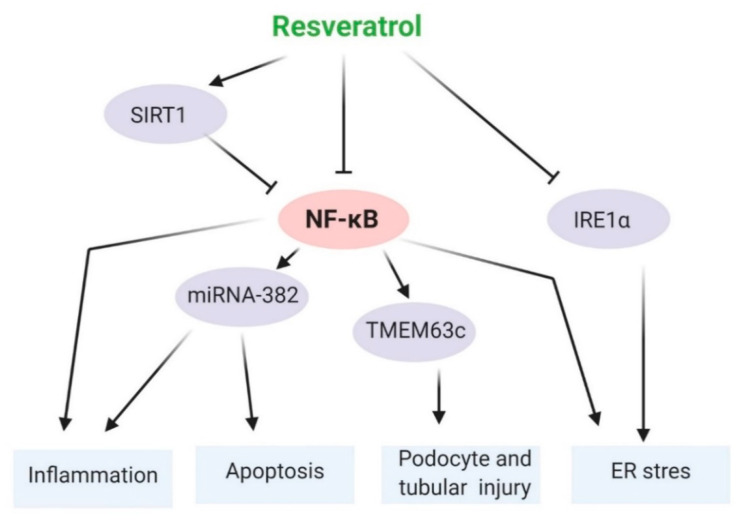
Resveratrol-inhibited NF-κB is associated with the inhibition of various mechanisms of age-related pathologies. Resveratrol inhibits NF-κB; thus, it may reduce kidney inflammation, tubular apoptosis and injury, podocyte apoptosis and damage, and ER stress. It also suppresses ER stress through inhibition of the IRE1α pathway. All of these pathways are involved in the higher potency of resveratrol against the NF-κB-associated aging process in kidneys. Membrane protein 63c, TMEM63c. Arrow means activation, and ‘T’ arrow means inhibition.

There are mainly three mechanisms involved in the anti-aging effects of resveratrol in the kidney, including SIRT1, AMPK, and the NF-κB pathways. These are discussed below and summarized in Figure 2, Figure 3 and Figure 4.

### 5.1. SIRT1 

SIRT2 overexpression may extend longevity in the yeast *Saccharomyces cerevisiae* by 30%, while suppression of the SIRT2 gene causes a reduction of its lifespan by almost half [160]. In mammals, CR induces SIRT1 activation, which regulates various physiological processes, such as mitochondrial fitness, metabolism, and the aging process, leading to an extension of lifespan [161,162,163]. In zebrafish, resveratrol mediates the AMPKα-SIRT1-PPARγ pathway and lipid metabolism [164]. In diet-induced obese mice, resveratrol improves metabolic phenotypes related to the aging process by downregulating cAMP and phosphodiesterases [165]. Thus, both resveratrol and CR have a beneficial effect in terms of metabolic regulation [166].

Several studies have suggested beneficial effects of resveratrol on kidney aging. A recent study showed that resveratrol protects against glomerulosclerosis in aged mice, improving kidney oxidative stress via SIRT1-mediated klotho expression [167]. Another study showed that resveratrol increased the SIRT1 expression level, which ultimately improves EMT and Yin yang 1 (YY1) acetylation induced by high glucose [168]. Resveratrol also reduces cadmium (Cd)-induced nephrotoxicity and mitochondria dysfunction through upregulation of VDAC1, Cyt C, SIRT3, SIRT1, PGC-1α, Nrf1, and TFAM [169]. The resveratrol-activated SIRT1 pathway plays a protective role by autophagy induction in I/R-induced AKI [170,171]. Resveratrol stimulates binding between forkhead box protein O (FoxO) 1 and SIRT1; thus, it may reduce kidney damage, myocyte hypertrophy, and interstitial fibrosis in nephrectomized mice [172]. The deacetylating activity of the resveratrol-activated SIRT1 pathway results in alterations in various downstream regulators, such as PGC-1α [173]. Elevated levels of NAD induced SIRT1, which leads to enhanced PGC-1α transcriptional activity [174]. Evidence from both in vitro and in vivo experiments suggests that this polyphenol can act as a safeguard for the kidney through maintenance of the SIRT1-PGC1α-FoxO pathway [88]. A recent study suggested that PGC-1α might be a potential therapeutic target against kidney aging [175]. Additionally, activation of SIRT1 by resveratrol treatment caused p53 deacetylation and thereby attenuated cisplatin-induced kidney injury and tubular apoptosis [52]. Resveratrol protects against Cd-induced nephrotoxicity by inhibiting IRE-1α and activating SIRT1 [129]. Kidney injury and fibrosis are attenuated by the resveratrol-induced SIRT1 signaling pathway through inhibition of EMT, where deacetylation of SMAD4 plays a vital role in inhibiting TGF-β and MMP7 [51,86]. Resveratrol attenuates ROS-induced oxidative stress through activation of the SIRT1 pathway [119]. Additionally, resveratrol treatment impedes high-glucose-induced cell senescence in the kidney by activating SIRT1 [113]. SRT1720 (an analog of resveratrol), an activator of SIRT1, reduces renal fibrosis by attenuating TGF-β1 and oxidative stress in UUO mice [176]. All of this evidence supports the fact that resveratrol may become a potential therapeutic against kidney aging through activation of SIRT1 and its target pathways (Figure 2). 

### 5.2. AMPK 

A potential role of AMPK signaling in kidney diseases, including diabetic nephropathy, polycystic kidney disease, subtotal nephrectomy, lupus nephritis, and kidney fibrosis, has been reported [177]. AMPK induces autophagy through upregulation of several antioxidants, such as SOD, uncoupling protein 2 (UCP2), and Nrf2, while it downregulates nicotinamide adenine dinucleotide phosphate oxidase (NOX, a primary source of ROS), suggesting a role of AMPK in the inhibition of oxidative stress in kidney disease [178,179].

In the process of aging, AMPK is the main nutrient sensor. Pro-longevity interventions, such as dietary restriction, induce AMPK activation to regulate cellular homeostasis [180,181,182]. The anti-aging effect of resveratrol is strongly linked to the activation of AMPK. Activation of AMPK is involved in lowering blood pressure in hypertensive mice [183]. In vascular smooth muscle, resveratrol promotes cellular differentiation through activation of the AMPK-SIRT1 pathway [184]. In primary human keratinocytes, resveratrol reduces oxidative stress-induced senescence by activating AMPK-FOXO3 [185]. AICAR, an activator of AMPK, increases the endogenous Sirt1 in mouse embryonic fibroblasts [186]. Resveratrol is an effective therapeutic agent against diabetic kidney fibrosis via AMPK/NOX4/ROS signaling [79]. Additionally, resveratrol alleviates age-related EMT in aging kidneys via AMPK-mTOR signaling [159]. All of this evidence suggests that resveratrol has significant effects on AMPK, which ultimately may help to fight against kidney aging (Figure 3). 

### 5.3. NF-κB 

Increased activity of NF-κB has been implicated in the pathogenesis of AKI [187]. Additionally, NF-κB promotes inflammation and regulates apoptosis; these two factors are associated with the progression of CKD [188]. In an in vivo study with mice, the upregulation of microRNA-382 in kidney epithelial cells was mediated by the activation of NF-κB signaling, which elevates pro-inflammatory cytokines [189]. Ang II and NF-κB play a vital role in podocyte injury via membrane protein (Tmem) 63c [190]. Furthermore, the pathogenic role of NF-κB in mediating chronic inflammation in tubular epithelial cells, podocytes, mesangial cells, and macrophages during CKD has been reviewed [191]. 

In a rat model of AKI, resveratrol increases the survival rate by promoting NF-κB-p65 deacetylation by upregulating SIRT1 and it inhibits inflammatory responses [192]. Resveratrol attenuates ER stress through suppression of IRE1 and NF-κB in kidney tubular cell injury [35]. Skeletal muscle atrophy is an important clinical characteristic of CKD. Resveratrol reduces skeletal muscle atrophy through suppression of NF-κB activation in in vivo models [42]. In addition, SRT1720 reduces vascular endothelial dysfunction by inhibiting aortic NF-κB activation and TNF-α levels in old mice [193]. In humans, resveratrol inhibits the signaling pathway of NF-κB, thereby inhibiting inflammation [194]. These studies suggest that through modulation of NF-κB pathways, resveratrol may act as a protective agent against kidney aging (Figure 4).

## 6. Resveratrol as Epigenetic Modulator 

Modulation of epigenetics is a major mechanism in aging [195]. Resveratrol mediates modifications to epigenetic enzymes, such as DNA methyltransferases (DNMTs), the histones acetyltransferases family (HATs), and the histone deacetylases family (HDACs), which ultimately impact our overall health and longevity [196]. While resveratrol increases AMPK, leading to activation of the SIRT1 pathway [164], SIRT1 catalyzes the deacetylation of histones and several transcription factors [197]. Thus, the beneficial effects of resveratrol are mediated through epigenetic modification by upregulation of the AMPK/SIRT1 pathway. SRT1720, a specific SIRT1 activator, mediates deacetylation and activation of PGC-1α, which restores tubular mitochondrial fitness, resulting in a decrease in I/R injury [198]. SIRT1 is suggested to deacetylate and inactivate the p65 subunit of NF-κB and STAT3, which reduces podocyte dysfunction in mice [199]. In addition, SIRT1 is found to deacetylate FoxO4 and it inhibits pro-apoptotic genes, such as Bcl2L11, which leads to a reduction in podocyte apoptosis [199]. SIRT1 increases the deacetylation of SMAD7, leading to inhibition of apoptosis in mesangial cells [200]. Resveratrol attenuates diabetic nephropathy through the activation of SIRT1 in rats. SIRT1 deficiency inhibits these effects [201]. Resveratrol protects the kidney by maintaining the SIRT1-PGC1α-FoxO pathway [88]. Histone H3.1 is a protein encoded by the *HIST1H3E* gene. In primary renal epithelial cells, epigenetic regulation of *HIST1H3E* has an overall effect on aging-related genes in humans. Additionally, resveratrol decreased *HIST1H3E* expression and increased *SIRT5* in muscle cells [202]. 

A recent review suggests that resveratrol mediates neuroprotective effects against Alzheimer’s disease pathology through epigenetic changes, including anti-aging effects in the brain [203]. Furthermore, another study has reviewed the epigenetic regulation of resveratrol against ocular diseases [204]. 

## 7. Resveratrol as a Calorie Restriction Mimetic

CR is an effective way of delaying the aging process and preventing chronic diseases, such as abdominal obesity, diabetes, hypertension, and cardiovascular diseases [205]. Glomerulosclerosis and kidney interstitial fibrosis occur in the aging kidney. It has been shown that long-term CR reduces aging-related kidney fibrosis by downregulating microRNA21 [206]. Short-term CR has potential in treating AKI [207]. Heat shock protein 47 (Hsp47) promotes kidney fibrosis and glomerulosclerosis in a rat model of CKD, while CR has been found to downregulate this protein, thus slowing the aging process of the kidney in mice [208,209]. Resveratrol mediates several mechanisms, such as activation of SIRT1, development of insulin sensitivity, and utilization of energy, which are closely related to the effects of CR. Resveratrol supplementation has exhibited beneficial effects by altering metabolic activities by improving insulin and glucose tolerance in old mice [166]. A randomized controlled trial revealed that among individuals with diabetes who take resveratrol, it helped them to decrease their level of fasting glucose, reduce their insulin resistance, and reduce their glycated hemoglobin levels [210]. A double-blind crossover study showed that resveratrol supplementation for a month in 50-year-old men with obesity mediated CR-like effects by inducing some changes in metabolism by regulating the AMPK–SIRT1–PGC-1α axis [211]. Several studies have proposed that resveratrol and CR have the same impact on various targets, such as adiponectin, AMPK, Akt, MnSOD, and NF-kB, in the cardiovascular system in mammals [212]. Apart from these, resveratrol and CR mediate similar effects against the aging of neuromuscular junctions and muscle fibers in old mice [213]. Accordingly, CR significantly decreases urea nitrogen, creatinine, and urine protein in CKD rodents [214]. Resveratrol-induced CR-like effects might alleviate age-related EMT in aging kidneys via AMPK-mTOR signaling [159]. It has been observed in genetically obese animals that decreased food intake prevents or partially delays some specific degenerative lesions, more specifically glomerulonephritis associated with obesity and diabetes [215]. In individuals suffering from type 2 diabetes with abdominal obesity, CR has shown improved glomerular hyperfiltration, and a similar effect has also been reported with the supplementation of resveratrol, suggesting that both CR and resveratrol can act as a protective agent against kidney aging [79,216]. Additionally, resveratrol has been shown to repress microRNA21 and NF-κB expression, leading to a decrease in pro-inflammatory cytokines, such as TNF-α, IL-1β, IL-6, and IL–8, while it downregulates MAPK, JNK, and AP-1 [217,218,219,220,221,222]. This evidence indicates that resveratrol may become a potential CR mimetic for improving kidney disease and aging.

## 8. Resveratrol in Lifespan Expansion 

Resveratrol is reported to increase the lifespan of many organisms. In *Saccharomyces cerevisiae*, it almost doubled its lifespan by stimulating SIRT2 activity [223]. Resveratrol also increases the lifespan of *Caenorhabditis elegans* (*C. elegans*) through SIRT2 activation without altering fertility [224]. Additionally, ad libitum addition of resveratrol to the diet may increase the lifespan of *Apis mellifera* [225]. Resveratrol extends the lifespan of *N. furzeri* along with a late decline in age-related brain action, particularly for motor and cognitive function [226]. In mammals, due to a lack of evidence, it cannot be said that there is a clear effect of resveratrol on lifespan extension. According to three different studies, resveratrol increases survival and insulin sensitivity, increases the lifespan, and improves locomotor activity; thus, it might be a potential treatment option against neurodegenerative diseases, as well as lengthening the lifespan, and it improves the function of motor neuron SOD1(G93A) in mice [227,228,229]. Resveratrol has shown protective effects against age-related kidney diseases by activation of SIRT1, an NAD(+)-dependent deacetylase, which may be a useful supplemental treatment for preventing age-related kidney injury [230]. Thus, these data suggest that resveratrol may also extend lifespan in the kidney.

However, a few studies support the fact that resveratrol treatment only slightly extends the lifespan of *C. elegans* and *D. melanogaster* and it had absolutely no effect on the crustacean model *Daphnia* [231,232]. A study on *D. melanogaster* also suggested that lifespan expansion solely depends on sex and diet [233]. Additionally, experiments with animal models showed that resveratrol administration for up to 1 year could not extend lifespan [234,235,236]. In aged mice, treatment with resveratrol delays age-related deterioration, including inflammation and oxidative stress, in vasculature and skeletal muscle [234,237]. A study found that resveratrol was associated with decreased survival rates in severe combined immunodeficiency in mice with prostate cancer xenografts [238].

## 9. Role of Resveratrol on Gut Dysbiosis and Associated CKD Pathobiology 

Gut microbiota plays a crucial role in immunity and inflammation [239]. Evidence for the existence of a gut–kidney axis suggests an intimate correlation between the abnormal gut microbiota and the development of CKD [240,241]. Dysbiosis in gut microbiota disrupts gut integrity and produces toxic metabolites, including urea and trimethylamine-N-oxide (TMAO), which lead to the aberrant activation of immune cells, excess production of inflammatory factors, and infiltration of inflammatory cells that can potentially contribute to CKD pathobiology [242]. 

Evidence shows that various polyphenols, including resveratrol, can promote gut microbiota by inhibiting various bacterial pathogens, namely *E. coli* and *Salmonella*, and thereby can improve inflammation and mitigate kidney damage [243]. Oral administration of resveratrol ameliorates gut dysbiosis in *db/db* mice by increasing the intestinal bacterial population, such as *Bacteroides, Alistipes, Rikenella, Odoribacter, Parabacteroides,* and *Alloprevotella*. Moreover, transplantation of fecal microbiota derived from healthy resveratrol-treated db/m mice was found to attenuate the renal dysfunction, rebalance the gut microbiome, and improve intestinal permeability and inflammation in recipient *db/db* mice [244]. In a study by Hu and colleagues, resveratrol was also shown to promote *Lactococcus lactis* but inhibit *Enterococcus faecalis* [245].

Resveratrol also can help improve other CKD-related risk factors, such as obesity, dyslipidemia, atherosclerosis, and CVD [246]. For example, resveratrol can modify the relative Bacteroidetes:Firmicutes ratio and reverse the gut microbial dysbiosis caused by a high-fat diet, and thereby promote energy metabolism to produce anti-obesity effects in rodents [247]. High concentrations of TMAO, a gut microbe-dependent metabolite of dietary L-carnitine and choline, are indicative of the development of CVD and CKD [248]. Dietary supplementation with resveratrol increased the abundance of *Lactobacillus*, reduced the levels of TMAO, and abrogated the atherosclerosis phenotype of ApoE-/- mice fed a high-choline diet [249]. Together, these findings show that resveratrol modulates gut microbiota and thereby plays a pivotal role in maintaining gut homeostasis and the prevention of CKD.

## 10. Prospects, Limitations, and Conclusions

**Figure 5:**
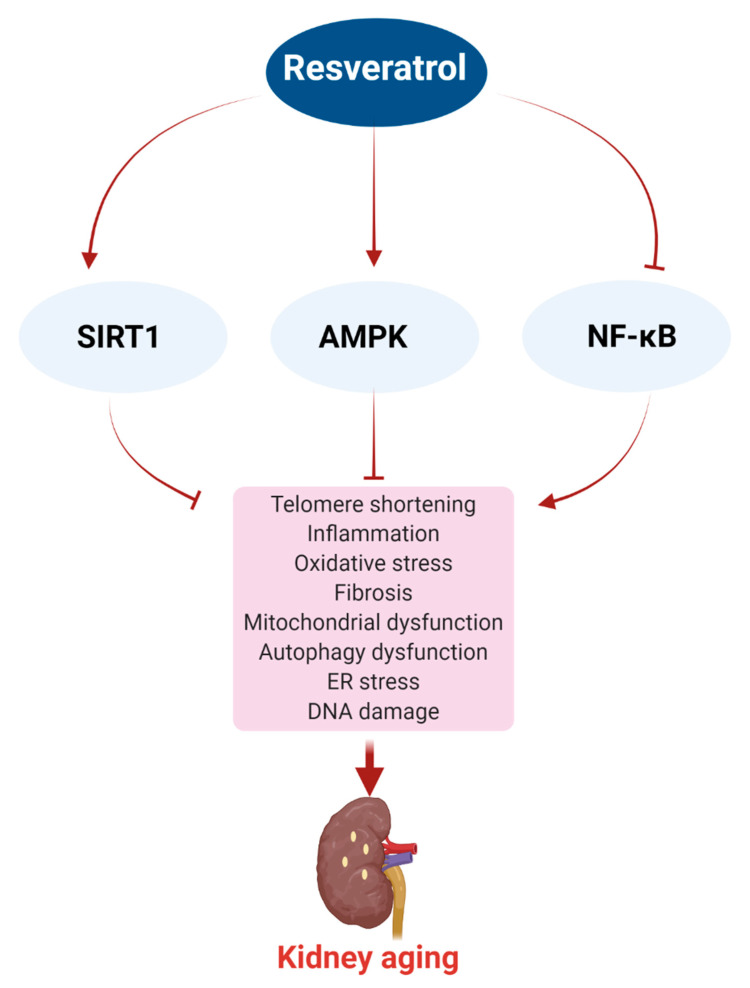
Potential anti-aging effects of resveratrol in the kidney. Resveratrol may mediate anti-aging effects in the kidney through the regulation of three main signaling pathways, SIRT1, AMPK, and NF-κB, which ultimately suppress pathological conditions, such as telomere shortening, inflammation, oxidative stress, fibrosis, mitochondrial dysfunction, autophagy dysfunction, ER stress, and DNA damage, in the kidney. Arrow means activation, and ‘T’ arrow means inhibition.

Kidney function decreases with age, and aging-associated kidney complications proportionately increase. Existing drugs for treating kidney diseases are limited by their side effects, and therefore natural compounds with fewer side effects are being evaluated. The literature highlighted in this review clearly suggests that resveratrol may modulate several pathological factors that are implicated in kidney aging, including inflammation, oxidative stress, fibrosis, mitochondrial dysfunction, cellular senescence, telomere shortening, ER stress, and autophagy dysfunction, and thus it may delay the aging process in the kidney (Figure 5). Aging biomarkers, such as SIRT1, AMPK, and NF-κB, and their associated signaling pathways are primarily targeted in resveratrol-mediated kidney protection. Moreover, resveratrol may increase the lifespan of model organisms and generate calorie restriction-mediated health effects, such as activation of SIRT1, development of insulin sensitivity, and utilization of energy.

While the pharmacological benefits of resveratrol in kidney aging have been revealed, these findings were mostly based on preclinical studies. The clinical applications of resveratrol are limited by its poor bioavailability and limited durability during delivery. Additionally, several studies have recognized resveratrol as a pro-oxidizing and cell-damaging agent [250,251]. A range of formulation techniques have been employed to overcome these difficulties [252]. Several nanoparticle-loaded resveratrol and kidney biomarkers are currently being investigated for efficient and stable drug delivery with substantial efficacy, as mentioned earlier [31,253]. Intensified clinical trials must be conducted to further evaluate its efficacy following by suitable strategies to achieve facile delivery in the human body.

We anticipate that the points discussed in this review will direct future research to better understand how pharmacological interventions through natural products could modulate kidney aging and help develop resveratrol as a potential anti-aging agent to manage aging-associated kidney abnormalities.

## Figures and Tables

**Table 1:** Pharmacological effects of resveratrol on different mechanisms involved in the aging process in various organs or cells or others.

Biological Sample Types	Experimental Models	Resveratrol Doses	Mechanisms Involved	Ref.
Blood plasma	Older adult humans	daily dose 1–2 g for 4 weeks	↑ Insulin sensitivity ↑ Plasma glucose in subjects with IGT	[60]
Blood plasma	Patients with peritoneal dialysis	150 or 450 mg/d	↑ Angiogenesis ↓ Ang-II	[61]
Brain	Ischemic brain in rats	40 μM/kg	↓ Lactate dehydrogenase ↓ Superoxide anion ↑ Mitophagy ↑ AMPK/autophagy ↓ Excess Ca^2+^	[62]
Brain	Traumatic brain injury in adult male rats	100 mg/kg	↓ IL-1β and IL-18 (inflammatory initiating cytokines) ↓ NLRP3 and caspase-1 pathways ↓ Inflammation and ROS ↑ SIRT1 activation	[63]
Heart	Type 2 diabetes and kidney hypertension in rats	5, 10 or 20 mg/kg/day for 4 weeks	↓ Serum MDA ↓ Systolic pressure, blood glucose, heart rate ↑ Serum SOD, glutathione reductase	[64]
Intestine and liver	Obesity in men	1000 mg/day for 1 week followed by 2000 mg/day for 2 weeks	↓ Intestinal as well as hepatic lipoprotein ↓ Apolipoprotein B (apoB-48) by 22%	[65]
Liver	HFD-induced fatty liver in mice	30 mg/kg/day	↓ TNF-a, IL-6, and IL-1b ↓ NF-κB pathway ↑ AMPKa ↑ SIRT1	[66]
Lung	Lung fibrosis in mice	50 and 100 mg/kg for 5 months	↓ NLRP3 ↓ Cytotoxicity ↓ IL-1β production ↓ inflammation and fibrosis	[67]
Lung	Nicotine-induced lung injury in rats	20 mg/kg/b.w. for 4 weeks	↓ IL-2, IL-6, TNF alpha, alpha-fetoprotein, plasma 8-hydroxydeoxyguanosine, Myeloperoxidase, XO, NO, lipid peroxidation ↑ Catalase, SOD, G6PD, and GSH-Px	[68]
Muscle	Barrows/male pigs	300 mg or 600 mg/kg of feed (dietary) for 49 days	↓ Muscle lactate, glucose ↓ MDA ↑ Crude protein and myoglobin content, ↑ T-AOC ↑ GSH-Px	[69]
Pancreas	Aged SAMP8 mice	5 mg/kg/day	↑ SIRT1 mRNA expression, ↓ NF-κB expression	[70]
Skin	UV-skin change in mice	10 µM /mouse	↓ Cellular proliferation ↓ Established markers of tumor progression (epidermal cyclooxygenase-2 and ornithine decarboxylase) ↓ Survivin expression	[71]
Mouse primary hepatocytes	NEFA-treated hepatocytes	100 µM	↓ TNF-a, IL-6, and IL-1b ↓ NF-κB pathway ↑ AMPKa ↑ SIRT1	[66]
Human epidermal keratinocytes	Foreskin biopsies-treated keratinoctyes	20 µM up to 100 µM	↑ Cellular glutathione content ↑ Nrf2 ↑ Cutaneous endogenous antioxidant status	[72]
Human nucleus pulposus cells	H_2_O_2_-treated pulposus cells	50 μM	↓ Mitochondrial dysfunction ↑ Autophagy	[73]
Human lung cancer cells (A549)	Non-small-cell lung cancer in cells	200 μM	↑ Beclin1 and LC3 II/I ↑ SIRT1 expression ↓ p62 expression ↑ Apoptosis ↑ Autophagy ↓ Akt/ mTOR pathway ↑ p-38-MAPK pathway	[74]
L6 rat skeletal muscle cells	2-deoxy-d-glucose-treated muscle cells	100 µM	↑ Glucose uptake in muscle ↑ Insulin action ↑ SIRT1, AMPK, GLUT4	[75]
Peritoneal mesothelial cells	High glucose in peritoneal dialysis solutions-treated cells	50 μM	↓ NLRP3 ↑ Autophagy ↑ AMPK-mediated autophagy	[76]

AMP-activated protein kinase, AMPK; angiotension II, ang-II; sirtuin type 1, glucose-6-phosphate dehydrogenases, G6PD; SIRT1; superoxide dismutase, SOD; glucose transporter 4, GLUT4; glutathione peroxidase, GSH-Px; malondialdehyde, microtubule-associated protein 1A/1B-light chain 3, LC3; MDA; mammalian target of rapamycin, mTOR; interleukin, IL; tumor necrosis factor alpha, TNF-α; nitric oxide, NO; non-alcoholic fatty liver disease, malondialdehyde, MDA; nuclear factor-κB, NF-κB; NLR family pyrin domain containing 3, NLRP3; impaired glucose tolerance, IGT; total antioxidative capacity, T-AOC; tumor necrosis factor-α, TNF-α, reactive oxygen species, ROS; non-esterified fatty acids, NEFA; ubiquitin-binding protein, p62; protein kinase B, Akt; and xanthine oxidase, XO; ↓: Decreased, and ↑: Increased.

**Table 2:** Pharmacological effects of resveratrol on the aging process in kidney tissues or cells.

Organs/Cells/ Tissues	Experimental Models	Resveratrol Doses	Mechanisms Involved	Ref.
Kidney	AKI in mice	100 μL of 100 mg/kg	↓ TLR4 activation, iNOS, ↓ Apoptotic factors (Bax, Bcl-xL)	[77]
Kidney	AKI in rats	30 mg/kg	↓ Serum creatinine and urea nitrogen levels, ↓ GRP78, Bip, pIRE1 and p65, TNF-α, IL-1β and IL-6 ↑ IL-10	[34]
Kidney	AKI in rats	30 mg/kg	↓ TNF-α, IL-1β and IL-6 ↓ pIRE1 and pNF-κB	[35]
Kidney	AKI in rats	100 mg/kg	↓ MDA and TNF-α ↑ GSH levels and SOD	[78]
Kidney	Cisplatin-induced kidney injury in mice	10 mg/kg	↑ SIRT1 and acetylation of p53 ↑ GFR	[52]
Kidney	*db/db* mice	40 mg/kg daily	↓ Serum creatinine, albumin, NOX4, αSMA, and fibronection; ↑ AMPK, and ACC	[79]
Kidney	Diabetic nephropathy in rats	(50 mg/kg/day)	↓ ER stress related factors (p-PERK, GRP78, ATF4, and CHOP)	[80]
Kidney	Diabetic nephropathy in mice	100 mg/kg /day for 12 weeks	↑ LC3-II/LC3-I and synaptopodin ↓ Cleaved caspase 3	[81]
Kidney	Diabetic nephropathy in rats	5 mg/kg/day	↑ SIRT1-mediated autophagy	[82]
Kidney	Hypertensive rats	50 mg for 9 weeks	↓ Kidney inflammation and injury ↓ Oxidative stress, ↑ Nrf2 and GST activity	[83]
Kidney	Progressive IgA nephropathy in mice	100 mg/kg	↓ NLRP3 inflammasome ↓ IL-1β, F4/80, CD3 ↓ Glomerular proliferation, glomerular sclerosis, and glomerular inflammation ↓ Superoxide anion levels	[84]
Kidney	Polycystic kidney in rats	200 mg/kg/day	↓ NF-κB (p50/p65) ↓ MCP-1, TNF-α, and CFB	[85]
Kidney	STZ-induced diabetes in rats	30 mg/kg/day	↓ Proteinuria, MDA, apoptosis ↑ Mn-SOD, SIRT1, PGC-1α	[55]
Kidney	STZ-induced diabetes in rats	30 mg/kg/day	↓ Renal function glomerulosclerosis, MDA, and acetylated-FOXO3a; ↑ SIRT1 deacetylase activity;	[50]
Kidney	UUO in mice	20 mg/kg	↓ Kidney injury & kidney fibrosis. ↓ MMP7, EMT	[86]
Kidney	UUO in mice	200 µg/g food	↓ NF-κB, IL-8, and TNF-α ↑ IL-10 and SIRT1	[51]
Kidney	UUO in rats	20 mg/kg/day	↓ MAPK, PI3K/Akt ↓ TGF-β1-induced FMD ↓ Myofibroblastic phenotype	[87]
Kidney	5/6 nephrectomy in rats	20 mg/kg	↑ Mitochondrial membrane potential and ATP ↑ SIRT1 and PGC-1α deacetylation	[43]
HK-2 cells	LPS-treated cells	20 μM	↓ TNF-α, IL-1β and IL-6 ↓ pIRE1 and pNF-κB	[35]
HK-2 cells	High glucose-treated cells	25 μM	↓ MDA, and acetylated-FOXO3a ↑ SIRT1 deacetylase activity;	[50]
Mouse podocytes	High glucose-treated cells	10 μM	↓ Mitochondrial ROS ↑ SOD, SIRT1, PGC-1α ↓ Apoptosis	[55]
NRK-49F cells	High glucose-treated cells	20 μM	↓ ROS, NOX4, αSMA, and fibronection; ↑ AMPK, and ACC	[79]
NRK-49F cells	Iohexol-treated cells	10 μM	↑ SIRT1, PGC-1α, SOD ↓ FoxO1, MDA	[88]

Acute kidney injury, AKI; AMP-activated protein kinase, AMPK; chronic kidney disease, CKD; glomerular filtration rate, GFR; sirtuin type 1, SIRT1; superoxide dismutase, SOD; glutathione peroxidase, GSH-Px; malondialdehyde, MDA; interleukin, IL; tumor necrosis factor alpha, TNF-α; nitric oxide, NO; nuclear factor- erythroid 2-related factor-2 (Nrf2); fibroblast growth factor 23 (FGF-23); glutathione-S-transferase, GST; NLR family pyrin domain containing 3, NLRP3; lipopolysaccharide, LPS; signal transducer and activator of transcription 3 (STAT3); forkhead box, FOXO; peroxisome proliferator-activated receptor-γ coactivator-1α, PGC-1α; caloric restriction, CR; monocyte chemoattractant protein 1, (MCP-1), Streptozotocin, STZ; tumor necrosis factor-α, TNF-α; and complement factor B, CFB, fibroblast–myofibroblast differentiation, FMD. ↓: Decreased, and ↑: Increased.

## Data Availability

Not applicable.
